# Characterization of TGFβ-associated molecular features and drug responses in gastrointestinal adenocarcinoma

**DOI:** 10.1186/s12876-021-01869-4

**Published:** 2021-07-12

**Authors:** Qiaofeng Zhang, Furong Liu, Lu Qin, Zhibin Liao, Jia Song, Huifang Liang, Xiaoping Chen, Zhanguo Zhang, Bixiang Zhang

**Affiliations:** 1 Hepatic Surgery Center, Tongji Hospital, Tongji Medical College, Huazhong University of Science and Technology, Hubei Province for the Clinical Medicine Research Center of Hepatic Surgery, 1095 Jiefang Avenue, Wuhan, 430030 China; 2 Hubei Province for the Clinical Medicine Research Center of Hepatic Surgery, Wuhan, 430030 Hubei China; 3 Hubei Key Laboratory of Hepato-Pancreato-Biliary Diseases, Tongji Hospital, Tongji Medical College, Huazhong University of Science and Technology, Wuhan, 430030 Hubei China; 4 Department of Anesthesiology, Union Hospital, Tongji Medical College, Huazhong University of Science and Technology, Wuhan, 430022 China

**Keywords:** Gastrointestinal adenocarcinoma, Transforming growth factor beta, Multi-omics signatures, Drug susceptibility, Deep neural network

## Abstract

**Background:**

Gastrointestinal adenocarcinoma (GIAD) has caused a serious disease burden globally. Targeted therapy for the transforming growth factor beta (TGF-β) signaling pathway is becoming a reality. However, the molecular characterization of TGF-β associated signatures in GIAD requires further exploration.

**Methods:**

Multi-omics data were collected from TCGA and GEO database. A pivotal unsupervised clustering for TGF-β level was performed by distinguish status of TGF-β associated genes. We analyzed differential mRNAs, miRNAs, proteins gene mutations and copy number variations in both clusters for comparison. Enrichment of pathways and gene sets were identified in each type of GIAD. Then we performed differential mRNA related drug response by collecting data from GDSC. At last, a summarized deep neural network for TGF-β status and GIADs was constracted.

**Results:**

The TGF-β^high^ group had a worse prognosis in overall GIAD patients, and had a worse prognosis trend in gastric cancer and colon cancer specifically. Signatures (including mRNA and proteins) of the TGF-β^high^ group is highly correlated with EMT. According to miRNA analysis, miR-215-3p, miR-378a-5p, and miR-194-3p may block the effect of TGF-β. Further genomic analysis showed that TGF-β^low^ group had more genomic changes in gastric cancer, such as TP53 mutation, EGFR amplification, and SMAD4 deletion. And drug response dataset revealed tumor-sensitive or tumor-resistant drugs corresponding to TGF-β associated mRNAs. Finally, the DNN model showed an excellent predictive effect in predicting TGF-β status in different GIAD datasets.

**Conclusions:**

We provide molecular signatures associated with different levels of TGF-β to deepen the understanding of the role of TGF-β in GIAD and provide potential drug possibilities for therapeutic targets in different levels of TGF-β in GIAD.

**Supplementary Information:**

The online version contains supplementary material available at 10.1186/s12876-021-01869-4.

## Introduction

Cancer of the digestive tract share a large quantity of global cancer incidences. gastrointestinal adenocarcinomas (GIADs), including esophageal adenocarcinoma (ESAD), stomach adenocarcinoma (STAD), colon adenocarcinoma (COAD), rectum adenocarcinoma (READ), revealed a nonnegligible global health burden in recent years [1]. Previous studies have established that all types of GIADs share the same features of DNA hypermethylation, mRNA expression and protein biomarkers, which confirmed GIAD in possession of exclusive characteristics [2, 3]. Apart from traditional histologic classification, an increasing number of molecular signatures were found across cancer. Models and clinical cohort were built to identify the function of molecular biomarkers. Thus, a dramatic increasing number of potential biomarkers were found and reported, which made clinical diagnosis, prognostic and immunotherapy more effective and reliable.

Transforming growth factor beta (TGF-β) family consists great many of activation and inhibition factors which participate in a variety of cellular biological processes [4]. As a prototypical factor in TGF-β family proteins, encoded by 33 genes in mammals, TGF-β is a multifunctional regulator involved in cell proliferation and differentiation [5], even in immune suppression within tumor microenvironment [6]. Some cell-surface transmembrane receptors with serine or threonine kinase activity can interact with activated TGF-βs, following phosphorylation of SMAD proteins, which then regulate the expression of TGF-β target genes [7]. Additionally, the activation of TGF-β signaling pathways can be negatively correlated with the development of COAD in multiple mechanisms [8]. And target genes of TGF-β are upregulated in ESAD samples while tumor progression is suppressed by TGF-β knockdown [9].

With the TGF-β signal transduction cascades being able to promote the growth and differentiation of tumor cells, and to inhibit cell proliferation in different tumor stages [4, 7, 10], it is still difficult to define the function of TGF-β signaling pathways in digestive tract adenocarcinomas. Moreover, TGF-β signaling targeted drugs have bright prospect in clinical application. Hence, more prospective research for TGF-β associated molecular signatures and possibility of targeted therapy in GIADs are in need.

We focused on multi-omics study across 4 cancer types in The Cancer Genome Atlas (TCGA) to elaborate different molecular pattern between TGF-β high expression (TGF-β^high^) and low expression (TGF-β^low^) cluster. An array of transcription, post-modification, proteomic change, gene mutation, genomic alteration and what regulating function they induced were analyzed in cancers. And the potential effects of anticancer drugs targeted on TGF-β signaling were assessed.

## Methods and materials

### TCGA and GEO data collection and processing

We collected multi-omics data (including count data of RNA-seq and miRNA-seq, Reverse phase protein lysate microarray (RPPA), mutation maf files and survival data) of 4 type of gastrointestinal adenocarcinoma (ESAD, STAD, COAD, READ) from Xena Hub. Genes with average expression of less than 1 in RNA-seq and miRNA-seq as well as synonymous mutations in the mutation data were filtered. Level 4 Copy number variation (CNV) data processed by GISTIC2.0 was obtained from Firehouse. Additional sequencing data for gastrointestinal adenocarcinoma were downloaded from NCBI GEO, including GSE19417, GSE62254, GSE17536, GSE45404, along with GSE62254 and GSE17536 contained survival data.

### Classification of TGF-β status in GIAD

39 core TGF-β associated genes were selected as TGF-β gene expression signatures, including TGF-β ligands, receptors, receptor substrates, Co-SMAD, inhibitory Smads, adaptors, and Smad chaperones. The specific details are as follows: 43 TGF-β core genes were selected from previous study [11], and the genes with low expression level were further removed from the expression data of TCGA's gastrointestinal adenocarcinoma (average count value less than 1), and 39 TGF-β core genes were finally obtained. In order to evaluate different TGF-β levels, two evaluation methods were used: (1) Unsupervised K-means clustering analysis based on the 39 mRNA of TGF-β-related signatures for each sample in GIAD; (2) Gene set variation analysis based on single sample gene-set enrichment analysis (ssGSEA) method was used to calculate the TGF-β score of each sample in GIAD. Unsupervised clustering was performed by R package ‘*ConsensuClusterPlus*’ [12], which is repeated 1000 times to ensure the stability of the results as in previous report [13].

### Analysis of alterations between different TGF-β status

For the mRNA, miRNA, and RPPA data in TGF-β^high^ and TGF-β^low^ group in each GIAD, we used the permutation test for differential analysis as previously described [14]. For RNA-seq, we further screened mRNAs with the absolute value of logFC (log_2_ (Fold change) = mean value in TGF-β^high^ / mean value in TGF-β^low^) > 1; for miRNA, we set the absolute value of logFC > 0.5 as the threshold; for RPPA data, we set the difference greater than 0.2 as the significantly changed proteins. For the mutation data, we selected high frequency mutations of more than 10% in the TGF-β^high^ or TGF-β^low^ group for analysis. Also, for CNV data, amplification or deletion regions exceeding 10% in the TGF-β^high^ or TGF-β^low^ group were selected for analysis. Fisher's exact test was used to evaluate significantly mutated genes, amplified and deleted chromosomal regions. We used FDR correction for all multiple tests, with FDR < 0.05 set as the threshold. Additionally, miRNA-targeted mRNAs were extracted from miRTarBase [15] and miRDB [16]. The mRNAs targeted by miRNA in two databases was intersected as the final targeted mRNAs. In order to analyze the correlation between miRNA and mRNA in different TGF-β status groups, principal component analysis (PCA) was used to reduce dimensionality to obtain the first principal component (PC1), and then Pearson correlation evaluation was conducted for the PC1. The advantage of this approach is that it concentrates on scores that are correlated (or inversely correlated) between gene sets.

### Enrichment analysis of pathways and gene set functions

We respectively performed Kyoto Encyclopedia of Genes and Genomes (KEGG) pathway analysis of the differentially expressed mRNAs in each type of GIAD, including genes upregulated in the TGF-β^high^ or TGF-β^low^ group. At the same time, gene-set enrichment analysis (GSEA) analysis was also used to analyze the significant up- or down-regulated pathways and functions in TGF-β^high^ group involved in each GIAD. Both KEGG and GSEA analyses are implemented on the R package ‘*clusterProfiler*’ [17]. The classic ‘Hallmark gene sets’ was selected as signatures for GSEA analysis. FDR < 0.05 was considered as significant enrichment.

### Analysis of drug-targeted TGF-β associated with gene signatures

The Pearson coefficient (*r*) between the expression of TGF-β specific mRNAs and the drug response (IC50) were evaluated in digestive tract cell lines. The drug response data of cell lines was downloaded from Genomics of Drug Sensitivity in Cancer (GDSC) [18]. Pearson correlation coefficient and FDR were calculated in GDSC, in which |*r*|> 0.3 and FDR < 0.05 were considered to be significant. We also further evaluated the estimated drug responses of 138 drugs in the TCGA sample from a previous study [19]. For these estimated drug responses, we used the Wilcoxon signed rank test, and FDR < 0.05 was considered to be differential.

### Construction of deep neural network (DNN)

Using genomic data, we trained a deep neural network (DNN) model to predict the TGF-β status of different GIAD datasets (Fig. 7a). In addition to the input layer, we construct a four-layer neural network, including three hidden layers and an output layer. We labeled the TGF-β^high^ group as 1 and the TGF-β^low^ group as 0. For a given hidden layer *l*, we use *ReLu* activation function. According to the output layer of the previous layer as the input of the next layer *A*^*[l−1]*^, we can carry out forward propagation as follows:

$$A^{\left[ l \right]} = \max \left( {W^{\left[ l \right]} A^{{\left[ {l - 1} \right]}} + b^{\left[ l \right]} ,0} \right),\left( {A^{\left[ 0 \right]} = X,l = \left( {1,2,3} \right)} \right)$$

where $A^{\left[ l \right]}$ is the output matrix of the hidden layer, $W^{\left[ l \right]}$ is the weight matrix, $b^{\left[ l \right]}$ is a column vector, and $X$ is the normalized matrix of input layer including the samples and gene signatures. Also, for the final output layer, we used the *sigmoid* activation function,

$$Y = \frac{1}{{e^{{A^{\left[ 3 \right]} }} + 1}}$$

where Y is the probability vector of the output layer, and $A^{\left[ 3 \right]}$ is the output matrix of the third hidden layer. We chose the loss function in logistic regression to measure the operation of the algorithm:

$$\begin{aligned}J\left( {W,b} \right) &= \frac{1}{m}\mathop \sum \limits_{i = 1}^{m} L\left( {\hat{y}^{\left( i \right)} ,y^{\left( i \right)} } \right)\\ &= \frac{1}{m}\mathop \sum \limits_{i = 1}^{m} \left( { - y^{\left( i \right)} log\hat{y}^{\left( i \right)} - \left( {1 - y^{\left( i \right)} } \right){\text{log}}\left( {1 - \hat{y}^{\left( i \right)} } \right)} \right)\end{aligned}$$

where $J\left( {W,b} \right)$ is the cost function, m is the sample number, $L\left( {\hat{y}^{\left( i \right)} ,y^{\left( i \right)} } \right)$ is the lost function of a sample, and $\hat{y}^{\left( i \right)}$ and $y^{\left( i \right)}$ are output value of DNN and the real label value of sample *i* respectively. Then, in order to minimize $\left( {W,b} \right)$, we used the stochastic gradient descent training model to learn $W^{\left[ l \right]}$ and $b^{\left[ l \right]}$. Learning rate (*α*) was set to be between 0.0005 and 0.005, and the epoch was set to be 100 to evaluate the best model. The *RMSprop* algorithm was used to optimize the model. TCGA data were randomly divided into training set and testing set according to 3:1. 75% of the TCGA samples were used for training, and the remaining 25% were used to test the performance of the model, which was evaluated by receiver operating characteristic (ROC) curve. Finally, we used this model to evaluate and analyze the TGF-β status on multiple GEO datasets.

### Other information for analysis

All analysis was done on R (Version: 3.6.1) or Python (Version: 3.6). Due to different types of survival data, survival analysis in TCGA refers to overall survival (OS) and progression-free interval (PFI), and in GEO datasets to OS and disease-free survival (DFS). In addition to the statistical analysis mentioned above, the log-rank test was used for survival analysis, and all multiple statistical tests were FDR corrected.

## Result

### Unsupervised classification of TGF-β status and overall patterns of multi-omics signatures between TGF-β^high^ and TGF-β^low^ groups

**Fig. 1:**
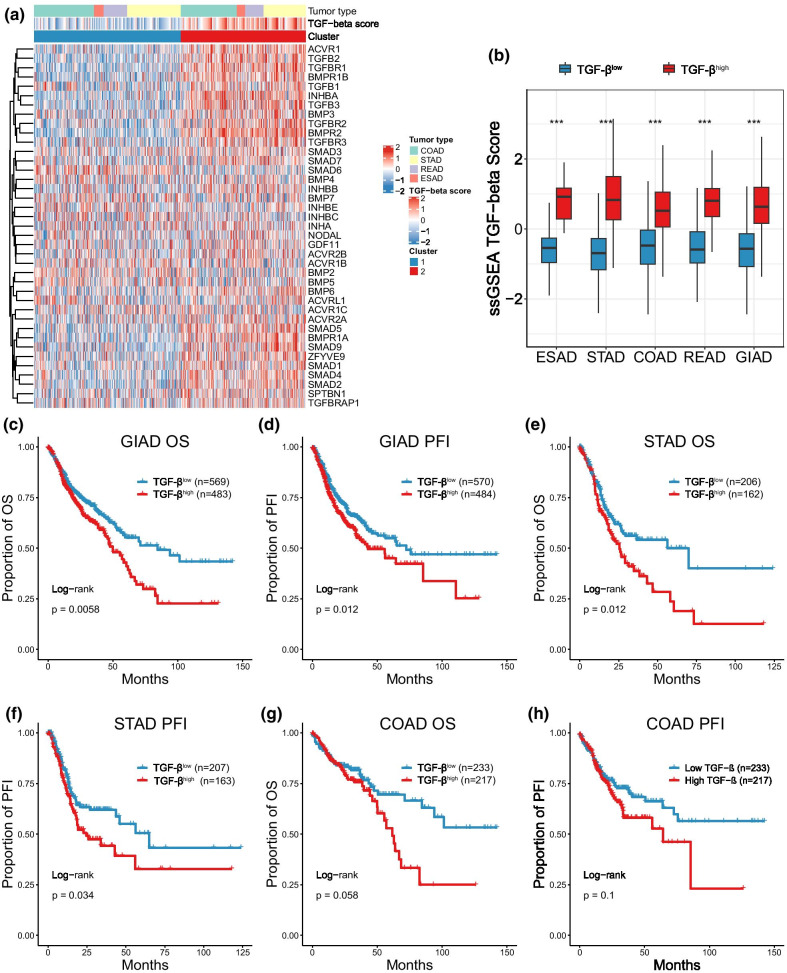
Unsupervised classification of TGF-β status and prognosis in gastrointestinal adenocarcinoma (GIAD). **a** Heatmap based on cluster of 39 TGF-β core genes. **b** Comparison of TGF-β scores in two clusters across different tumors. **c–h** Kaplan-survival curves (including overall survival and progression-free interval) for different TGF-β groups in GIAD. *P < 0.05, **P < 0.01, ***P < 0.001

Unsupervised clustering was used to identify the activation of respective driver genes between two clusters characterized by low and high distribution of TGF-β core genes (Fig. 1a). We used single-sample gene set enrichment analysis (ssGSEA) to further exhibit that cluster2 had higher TGF-β scores not only in various categories of GIAD but also in general (Fig. 1b). Correlation analysis was performed to exhibit that TGF-β core genes were correlated to TGF-β score in a positive tendency (Additional file 1: Fig. S1a), which explained the consistency of gene activation and cluster outcome. Therefore, we set cluster1 as the TGF-β^low^ group and cluster2 as the TGF-β^high^ group.

To analyze the prognosis of different TGF-β groups, we performed a survival analysis. As a whole, GIAD patients with high activation of TGF-β pathway had statistically significant poor prognosis (OS: HR = 1.374, 95% CI 1.095–1.723, *P* = 0.0058; PFI: HR = 1.329, 95% CI 1.065–1.659, *P* = 0.012) (Fig. 1c, d). Specific to each tumor, although only STAD showed a significant difference between two groups (Fig. 1e, f), a trend of poor prognosis still shown in TGF-β^high^-specific COAD and ESAD patients (Fig. 1g, h, Additional file 1: Fig. S1b–e), which partially elucidated the causality of poor prognosis and TGF-β pathway activity.

**Fig. 2:**
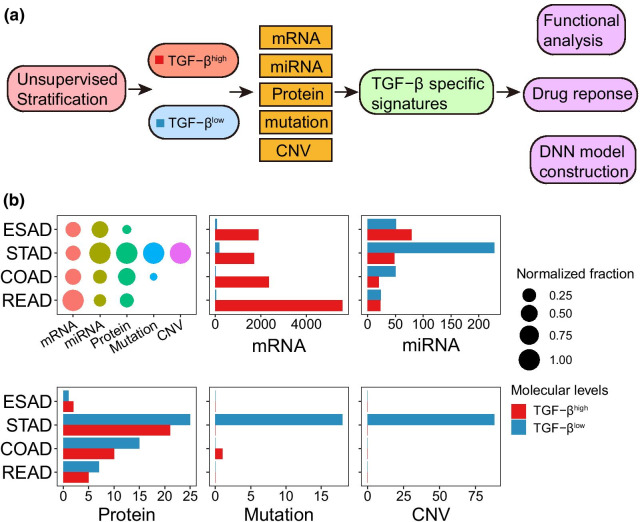
TGF-β specific multi-omics signatures in GIAD. **a** Overview of analysis process in this study. **b** The relative abundance and the number of each molecular signature in TGF-β^high^ and TGF-β^low^ group. The dot plot represents the proportion of molecular characteristics of each tumor in all GIADs, and the bar plot represents the number of molecular signatures of each tumor in the TGF-β^high^ and TGF-β^low^ group (FDR < 0.05)

To further investigate TGF-β associated molecular features across GIAD, a procedure was designed with analytic pipeline outlined in Fig. 2a. Then, we analyzed various molecular signatures between previous cluster groups, including mRNA expression (approximately 18,000 genes), miRNA expression (approximately 600 miRNAs), protein expression (198 proteins), somatic mutation (frequency > 5% in at least one cluster) and copy number variation (CNV) (Additional file 2). And the most striking signatures turned out to be mRNA, miRNA and protein expression (Fig. 2b). The number of mRNA genes ranged from 1865 in STAD to 5606 in READ, most of which surprisingly belonged to high score group. The number of miRNAs showing significant expression level ranged from 47 in READ to 276 in STAD. Alterations of protein expression exhibited the largest number in STAD, and significant alterations of low score group were more than high score group across cancer types. However, the differences in protein and mRNA expression between the two groups were not consistent. The possible reason is that, unlike the sequencing of all the genes encoding the transcriptome, only 198 classical proteins were included in the study in the sequencing of proteins, and there were a lot of post-transcriptional modifications in these classical proteins, including phosphorylation, ubiquitin, glycosylation and the SUMOylation, which would affect the overall difference between mRNA and protein. We also found that STAD had a large amount of alterations in somatic mutation and CNV, while both clusters of other cancer types showed non-differential signatures. In aggregate, STAD got the most TGF-β associated signatures, including 1865 mRNA genes, 276 miRNAs, 46 proteins, 18 gene mutation and nearly 90 CNV regions.

### TGF-β effects on mRNA and functional pathways

**Fig. 3:**
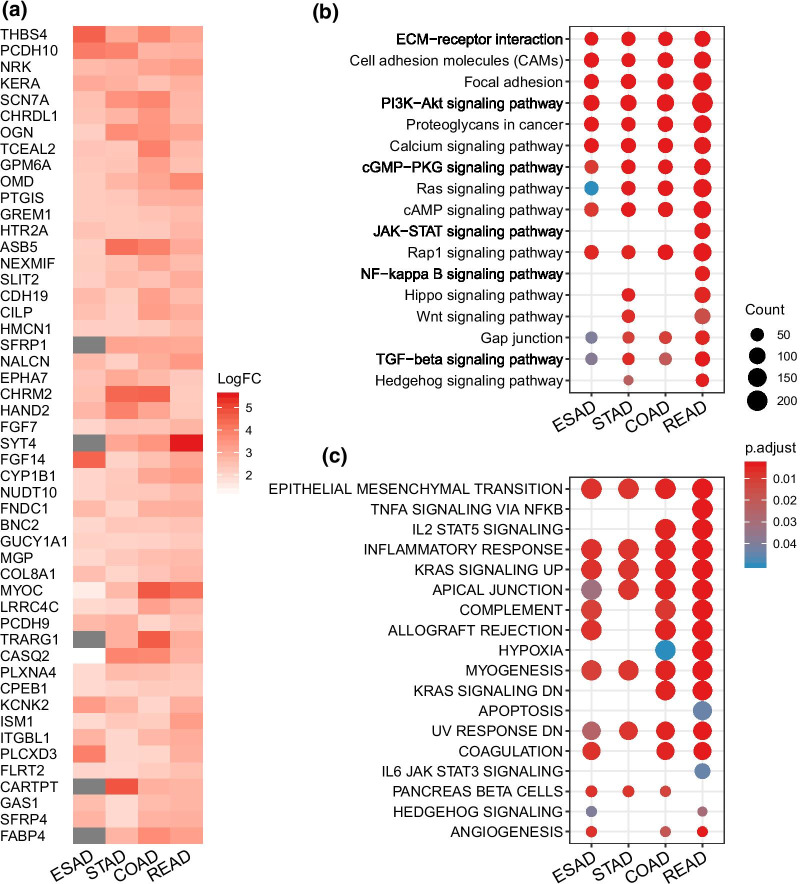
TGF-β effects on mRNA and functional pathways. **a** The heatmap shows the most significantly upregulated 50 mRNAs in the TGF-β^high^ group across each GIAD. **b** KEGG pathways [54] enriched by significantly upregulated mRNA in the TGF-β^high^ group (FDR < 0.05). **c** Overall gene-set enrichment analysis (GSEA) pathway enrichment analysis was performed for the differentially expressed mRNAs (TGF-β^high^/TGF-β^low^)

To understand the function of TGF-β associated mRNA signatures in digestive tract adenocarcinoma, we compared mRNA expression levels for each gene. Then a robust rank aggregation (RRA) analysis was performed to rank top 50 genes with the highest expression in TGF-β^high^ group as previous research did [20] (Fig. 3a). For example, thrombospondin 4 [21] (THBS4), gremlin 1 [22] (GREM1), slit guidance ligand 2 [23] (SLIT2) and hemicentin-1 [24] (HMCN1, also known as fibulin-6) was able to mediate the function of cell proliferation, invasion and migration induced by TGF-β1 (Fig. 3a). Enriched KEGG pathway of differentially expressed mRNAs revealed that genes involved in extracellular matrix signaling, cell adhesion, classic PI3K/AKT signaling pathway and proteoglycans expression were up-regulated in high TGF-β score samples (Fig. 3b). Meanwhile, due to the small number of down-regulated genes in the TGF-β^high^ group across cancer types, there were fewer common genes and enriched pathways in the four tumors, with the result of pathway enrichment focused on ESAD and STAD (Additional file 1: Fig. S2a–b). To further manifest the enriched functions of differentially expressed mRNAs, we performed gene set enrichment analysis (GSEA) on all of them (Fig. 3c). Some of the enriched functions across cancer types were associated with TGF-β induced biological process, such as epithelial mesenchymal transition (EMT) and inflammatory response, which was involved in tumor genesis and metastasis [6, 25]. We also noticed that signaling pathways of immune regulation and cellular metabolism were significantly enhanced. In general, the results were unsurprisingly consistent with previous findings. It is noteworthy that the EMT showed the most significant enrichment in our analysis, suggesting that TGF-β signaling pathway in GIAD may promote the occurrence of EMT and tumor metastasis, which is also consistent with previous research reports [26].

### Comparison of miRNA, and protein expression in different TGF-β status

**Fig. 4:**
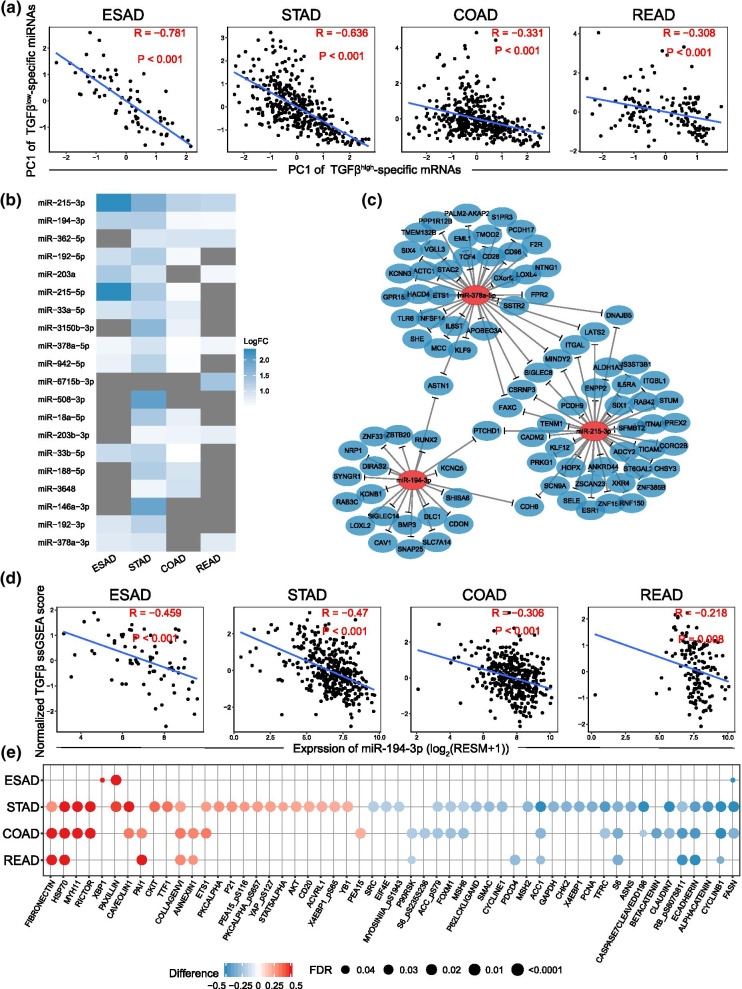
Comparison of miRNA, and protein expression in different TGF-β status. **a** Correlation between the first principal component (PC1) of the expression of TGF-β^low^-specific miRNAs and their predicted targeted TGF-β^high^-specific mRNAs. **b** The heatmap shows the most significantly down-regulated 20 miRNAs in the TGF-β^high^ group across each GIAD. **c** A network Schematic diagram of miR-215-3p, miR-378a-5p, and miR-194-3p and their predicted targeting TGF-β^high^-specific mRNA in stomach adenocarcinoma. **d** Negative correlation between miR-194-3p expression and TGF-β score in 4 types of GIAD (Pearson correlation). **e** The significantly differential proteins in TGF-β^high^ and TGF-β^low^ groups; Red indicates high protein expression in the TGF-β^high^ group, and blue indicates high protein expression in the TGF-β^low^ group

As we all known that miRNA is the key regulator in post-transcription of gene expression [13]. In our study, differentially expressed miRNAs were detected across cancer types. Principal component analysis and correlation analysis were implemented to describe the correlation in between differentially expressed miRNAs and their predicted downstream regulation targets, mRNAs. And the results testified a negative regulation function of high expressed miRNAs in TGF-β^low^ group (TGF-β^low^ specific miRNAs) by showing a significant negative correlation for 4 types of cancers (Fig. 4a). Meanwhile, the same trend was also found in TGF-β^high^ specific miRNAs with low expression and its targeted mRNAs in TGF-β^high^ group (Additional file 1: Fig. S3a). These indicated that TGF-β associated mRNAs are regulated by the associated TGF-β specific miRNAs.

Subsequently, we arranged the top 20 reduced miRNAs, with 3 (miR-215-3p, miR-378a-5p, and miR-194-3p) of them significantly downregulated in TGF-β^high^ cluster across cancer type (Fig. 4b). Specifically, miR-215-3p was reported to be a co-inhibitor of cell migration induced by TGF-β1 [27]. And the overexpression of miR-194-3p significantly inhibited RUNX2 [28] and PRC1 [29] signaling pathway which was crucial to cell proliferation and migration. Nevertheless, the effects of miR-378a-5p on cell proliferation, migration and invasion were controversial according to recent studies [30, 31]. The network of these representative miRNAs and enriched target mRNAs were exhibited in Fig. 4c. The expression level of miR-215-3p, miR-194-3p and miR-378a-5p were negatively correlated with TGF-β pathway scores across cancer types, which substantiated performed results (Fig. 4d, Additional file 1: Fig. S3c–d). Though the regulatory relationships were not clear between TGF-β^high^-specific expressing miRNAs and corresponding mRNAs, which might owe to few samples of TGF-β^low^-specific mRNAs (Additional file 1: Fig. S3a–b). A recent research suggested that expression of miR-100 and miR-125b, which got involved in EMT, tumorigenesis and poor prognosis, was induced and upregulated by TGF-β [32].

Standardized differences of protein expression levels between two groups were displayed in Fig. 4e. And we focused on STAD, COAD and READ due to unapparent alterations in ESAD samples. For example, downregulated E-cadherin in TGF-β^high^ group suggested its inhibiting effect of metastasis in cancer, which was concordant with previous studies [33, 34]. On the contrary, collagen IV [35] and fibronectin [36] were reported as pro-metastasis factors and found unsurprisingly overexpressed in TGF-β^high^ group. We also found that proteins associated with cell cycle (such as cyclinb1 and cycline1) were downregulated in TGF-β^high^ cluster, which exactly verified the inhibitory effect of TGF-β on proliferation [4].

### Significant mutations and CNVs between the TGF-β subgroups in GIAD

**Fig. 5:**
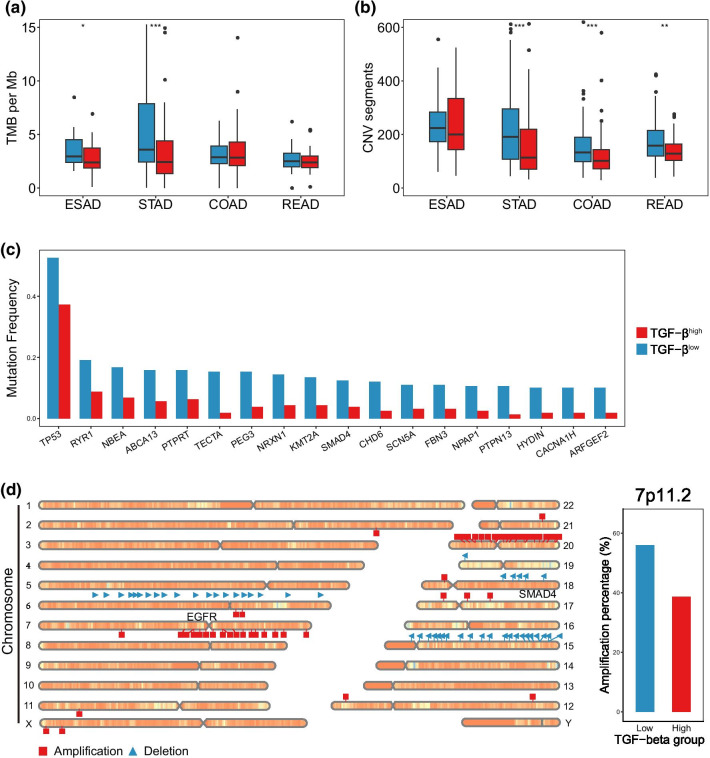
Significant mutations and CNVs between the TGF-β subgroups in GIAD. **a, b** Comparison of tumor mutation burden (TMB) and CNV burden in different TGF-β cluster. **c** Mutation frequency of significant mutation gene in two TGF-β subtypes in stomach adenocarcinoma (Fisher’s exact test, FDR < 0.05). **d** Chromosome map shows significant amplification and deletion of chromosome regions in stomach adenocarcinoma (Fisher’s exact test, FDR < 0.05); The bar plot on the right panel shows the amplification of the chromosomal region where EGFR is located. *P < 0.05, **P < 0.01, ***P < 0.001

Various patterns of TGF-β signaling were historically found to be associated with genomic stability and gene expression in gastroenteric tumor [37]. Therefore, we analyzed the differences between low and high TGF-β expression at the genomic level. Overall, poor genomic stability was found in TGF-β^low^ cluster on account of a higher level of tumor mutation burden (TMB) and CNV, with STAD the most significant one across cancer types (Fig. 5a–b).

Subsequently, we focused on significant gene mutations in individual tumors, and only in COAD and STAD did we find significant mutations (FDR < 0.05). In addition to more EP300 mutation in the TGF-β^high^ group in COAD, we found more interesting results in STAD. Specifically, there were a total of 18 mutated genes with significant difference in 2 STAD subtypes, all of which had more high-frequency mutations in the TGF-β^low^ group (Fig. 5c). As an outstanding tumor suppressor gene activated by oncogenic stress, TP53 mutation occurred frequently in various tumors [38], and showed the highest frequency of 52.6% in our study. Recent studies identified a high correlation between TP53 mutation and tumor immune response [39] with relevant TGF-β signaling induced immune evasion and immunotherapeutic resistance [40]. Significant CNVs of STAD in TGF-β^low^ cluster showed that amplification and deletion of genes were concentrated on several chromosomes (Fig. 5d). In particular, EGFR from region 7p11.2 amplified more frequently in TGF-β^low^ samples (56.0%) leading to an increased sensitivity to targeted therapy [41, 42]. Whereas SMAD4 from 18q21.2, as a key mediator of TGF-β signaling pathway, exhibited a significant deletion (52.6%).

### Analysis of TGF-β associated gene signatures on drug susceptibility in GIAD

**Fig. 6:**
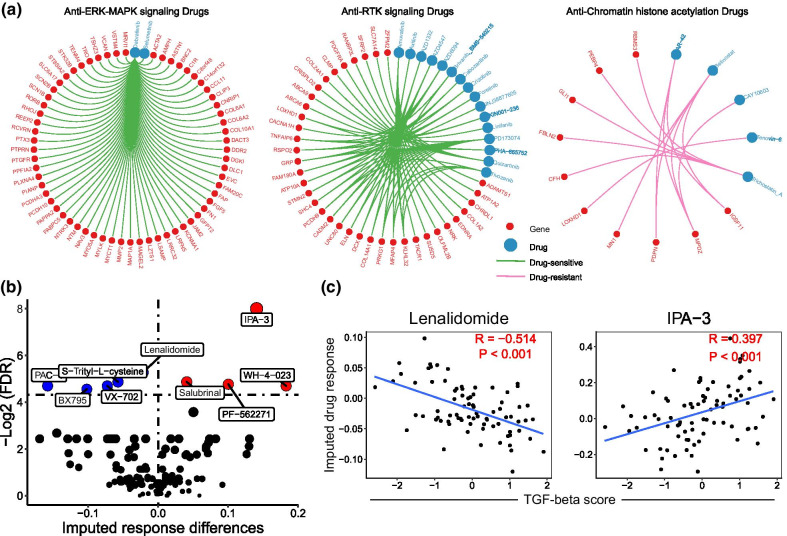
Analysis of TGF-β associated gene signatures on drug susceptibility in GIAD. **a** TGF-β associated gene responses to drugs involved in different pathways; The red dots represent genes, the blue dots represent drugs, the green line indicates high gene expression is sensitive to drugs, and the pink line indicates high gene expression is resistant to drugs. **b** Volcano plot showed imputed drug response differences in the TGF-β subgroups in GIAD (most samples are ESAD samples); The blue dots indicate significant drug-sensitivity, and the red dots indicate significant drug-resistance (FDR < 0.05). **c** The correlation between lenalidomide (or IPA-3) and TGF-β score

We wanted to further investigate the TGF-β signaling specific sensitivity of targeted drugs in digestive tract adenocarcinoma. By identifying a total of 760 differentially expressed mRNA genes associated with TGF-β, we used Pearson correlation test between gene expression and 50% inhibiting concentration (IC50) for 345 anticancer drugs to assess drug response across 106 digestive tract cancer cell lines from the Genomics of Drug Sensitivity in Cancer (GDSC). We focused on these significant correlations between 311 genes and 73 drugs, targeting on ERK, JNK and p38 signaling, RTK signaling, chromatin signature, cell cycle, apoptosis and PI3K/mTOR signaling (FDR < 0.05, Fig. 6a and Additional file 1: Fig. S4a–h). And dabrafenib got the largest quantity of associated genes (62 genes), such as FAP, FN1 and MMP2 (r_FAP_ = − 0.51, r_FN1_ = − 0.42, r_MMP2_ = − 0.51), which promoted cell invasion, metastatic and EMT [43–45]. Then we calculated imputed tumor response as quantified sensitivity of 138 anticancer drugs in 79 TCGA patient samples (mainly ESAD) to assess the association between drug response and activation level of TGF-β signaling pathway. As a member of mutant BRAF-V600 inhibitors, debrafenib was reported be one favourable option for BRAF-V600 mutant malignances [46]. BRAF is one high frequency mutated kinase in human cancer and V600 a canonical mutation site in conserved domain, suggesting a significant ultilizing on clinical treatment and prognosis prediction. In melanoma, activation of TGF-β signaling directly upregulates the expression of EGFR and platelet-derived growth factor receptor-β, which leads to BRAF inhibitor resistance [47]. And BRAF-V600 inhibitor can suppress the activation of TGF-β signaling via dephosporylation of TFEB, while TFEB phosphorylation and TGF-β upregulation play a pro-tumorigenic role in BRAF inhibitor resistant melanoma [48]. Our analyzed enriched genes associated with debrafenib provides a way to further understanding of underlying mechanism. However, the application of BRAF-V600 inhibitors in the treatment of digestive tract adenocarcinoma is almost limited to colorectal cancer. Our result suggests the clinical potential of BRAF-V600 inhibitors in other GIACs. Significant imputed response differences of anticancer drugs were identified with 4 of them resistant but 5 of them sensitive to digestive tract adenocarcinoma (Fig. 6b). In our study, correlations between TGF-β score and drug sensitivity that were most significant were exclusively analyzed with a positive result for lenalidomide (r = -0.51) but quite opposite for IPA-3 (r = 0.40) (Fig. 6c).

### Identification of two TGF-β subtypes based on the deep neural network (DNN) associated with TGF-β signatures

**Fig. 7:**
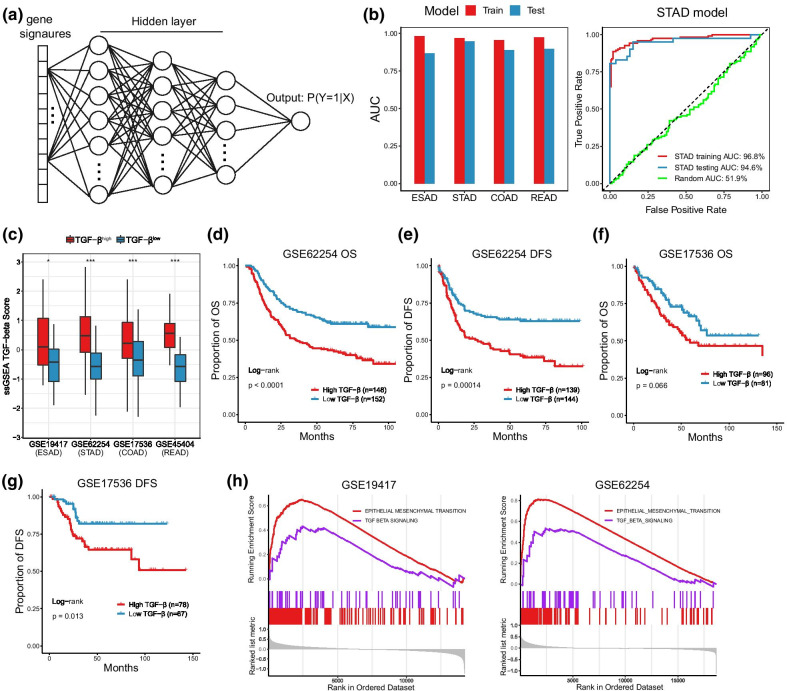
Identification of two TGF-β subtypes based on the deep neural network (DNN) associated with TGF-β signatures. **a** Schematic diagram of a deep neural network for predicting TGF-β subtypes. **b** The performance of our DNN classifier on training and testing sets; Left: area under roc curve (AUC) of receiver operating characteristic (ROC) curve across each tumor. Right: ROC curve of the DNN classifier in stomach adenocarcinoma. **c** TGF-β score in different TGF-β subtypes for each other dataset classified by the DNN model. **d–g** Kaplan-survival curves (including overall survival and disease-free survival) for different TGF-β groups identified by DNN model in GSE62254 and GSE17536. **h** Gene-set enrichment analysis showed that TGF-β and EMT pathways were significantly enriched in different TGF-β groups identified by DNN model. *P < 0.05, **P < 0.01, ***P < 0.001

In order to better examine the application of TGF-β associated signatures in the classification of TGF-β subtypes, we developed a DNN model to identify TGF-β subtypes (Fig. 7a). The advantage of DNN is that it can extract the characteristics of the data as much as possible and classify the nonlinear data. We used the aforesaid top 10 gene signatures with smallest p value in Fig. 3a as input data, which could which can fully represent the characteristic information of different groups of TGF-β. Through the GIAD data marked in TCGA, we trained in 75% of the data and tested in the remaining data. We found that in TCGA, our model was able to distinguish between the groups with TGF-β^high^ and TGF-β^low^ status. Overall, the DNN classifiers demonstrated excellent performance, with the area under roc curve (AUC) of receiver operating characteristic (ROC) curve across all tumors above 86% in both the training set and the test set. For example, in STAD, AUC was 96.8% and 94.6% in training and test sets, respectively (Fig. 7b).

Further, we analyzed other GIAD datasets. In the other datasets of four types of GIAD, we used the DNN model to identify TGF-β subtypes. Obviously, TGF-β scores were higher in the DNN-identified high TGF-β^high^ group (Fig. 7c). Meanwhile, we evaluated prognosis in two of the datasets (GSE17536 (COAD) and GSE62254 (STAD)) with survival data. In both datasets, the TGF-β^high^ group had a poorer prognosis, including OS and DFS (Fig. 7d–g), although there was no statistical difference in OS for GSE17536 (P = 0.066). Finally, we performed GSEA analysis for all TGF-β subgroups identified by DNN model in four types of GIAD, in which EMT and TGF-β signaling pathways were significantly enriched (all P < 0.05, Fig. 7h, Additional file 1: Fig. S5). These results indicate that our DNN model is a good predictor of TGF-β status and patient prognosis in COAD and STAD.

## Discussion

As a large and classic family of molecules, TGF-βs induce a series biological processes involved in tumorigenesis and metastasis, which can be sensitive to targeted drug therapy. The main function of TGF-β signaling pathway varies from different cancer types and different stages of cancer. First of all, we performed an unsupervised clustering to characterized expression status of TGF-β signaling and associated molecular differences in each cancer type. By identifying the relevancy between TGF-β high expression and poor prognosis, our method used permutation test to implement multi-omics variation analysis. In this way, we managed to comprehensively evaluate the functions of TGF-β signaling by analyzing differentially expressed miRNAs and its negatively regulated mRNAs. In the meantime, identified gene mutations and CNVs demonstrated genetic approaches to a wide range of biological processes, such as cell cycle, angiogenesis, EMT, cell invasion and other downstream pathways. Considering that TGF-β associated gene and protein expression get involved in cancer progression (metastasis in especial), we then used the method from a previous study to identify drug response targeted at aforesaid signatures for further research of targeted therapy [14]. Several drugs suggested a great sensitivity to biological processes of tumor cells induced by TGF-β and related molecules. Moreover, some classic oncogenes were found to be targeted by dozens of anticancer drugs, which improved the feasibility of targeted therapy. Also, based on TGF-β-specific transcriptional signatures, we developed a deep neural network classifier that could well predict TGF-β levels in different datasets of GIAD.

In our study, TGF-β^high^ patients displayed a high propensity of overexpressed mRNAs which are relevant to extracellular matrix organization and cellular biological process. These cell behaviors included proliferation, adhesion, invasion and migration, which can be inhibited by regulations at transcriptional level for TGF-β^low^ patients. We also noticed that a number of metastasis promoting proteins (such as collagen IV, fibronectin) were upregulated in TGF-β^high^ samples with inhibiting proteins (such as E-cadherin, FASN) downregulated. In line with our finding, a previous research comprehensively identified the function of TGF-β family in both transcriptional and molecular level [5]. Genomic analysis led to an impressed result that occurrence of significant alterations was tendentious across multiple cancer types (especially STAD). It is worth noting that in our mutation and CNV analysis, both significant mutation and CNV loci were present only in STAD; however, similar trends were observed in other gastrointestinal adenocarcinomas (Additional file 1: Fig. S6), but there was no statistically significant difference. However, in the overall analysis, there was more mutation load and copy number load in the TGF-β^low^ group (Fig. 5a, b), which indicated the consistency of gastrointestinal adenocarcinoma to some extent. Specifically, a high level of TMB and SCNA lead to modification of coding proteins in TGF-β^low^ samples, which can activate abundant anti-tumor immune response as tumor-specific neoantigens for TGF-β^low^ GIADs. Recently, immune responses were found to be suppressed due to TGF-β induced abnormal function of immune cells within tumor microenvironment [6, 49, 50]. As for our observation, immune evasion induced by high TGF-β expression and lack of immune response in TGF-β^high^ GIAD interpreted the poor prognosis of TGF-β^high^ patients, which was just the opposite situation in TGF-β^low^ group.

Our analysis about drug sensitivity revealed that differentially altered gene were targeted by anticancer drugs for various clinical therapies. The most representative drug dabrafenib, inhibitor of BRAF-V600 mutation, is sensitive to genes related to cell invasion, metastatic and EMT, such as FAP, FN1 and MMP2. We predict dabrafenib one potential anticancer drug for GIAD patients. Lenalidomide, an immunomodulator, can facilitate activation and function of immune cells and participates in induction of tumor cell apoptosis. Due to imputed response differences, lenalidomide may be a valid anticancer drug for TGF-β^high^ patients. Though several trials of targeted drugs for TGF-β associated signatures have been implemented and reviewed, the molecular mechanisms of targeted drugs remained unspecific [7, 51–53]. The role of clinically actionable genes in cancer progression and metastasis deserves further explanation so that rational combination therapies can be exploitable to promote prognosis in cancer patients. Hence, our systematic identification of TGF-β molecular signatures and sensitivity analysis can benefit clinical targeted therapies which are expected to be more efficient in the future.

This study has certain limitations. Despite these causalities and correlations elaborated by our and many previous studies, further experimentations and analyses can be helpful to extend these conclusions and clinical viability. Retrospective studies and large-scale clinical trials are necessary for experimental verification. Finally, our combination of multi-omics is comprehensive but not exhaustive. Potential regulatory pathways and molecular mechanisms need further investigation. In addition, prospective cohort studies are called to manifest clinical benefits for patients with tumor.

## Conclusions

Our study provided a comprehensive analysis of the molecular characteristics associated with TGF-β and provides possible therapeutic targets in GIAD.

## Supplementary Information

**Additional file 1**. Supplementary figures.

**Additional file 2**. Molecular characterization of TGF-beta in GIACs.

## Data Availability

Data in this study are from the TCGA, GDSC, and GEO database. To view additional sequencing data involved in this article please visit websites at: https://www.ncbi.nlm.nih.gov/geo/query/acc.cgi?acc=GSE19417, https://www.ncbi.nlm.nih.gov/geo/query/acc.cgi?acc=GSE62254, https://www.ncbi.nlm.nih.gov/geo/query/acc.cgi?acc=GSE17536, https://www.ncbi.nlm.nih.gov/geo/query/acc.cgi?acc=GSE45404, https://www.ncbi.nlm.nih.gov/geo/query/acc.cgi?acc=GSE62254, https://www.ncbi.nlm.nih.gov/geo/query/acc.cgi?acc=GSE17536. To view multi-omics data of ESAD, STAD, COAD and READ please visit websites at: https://xenabrowser.net/datapages/. Data downloading and processing are as described in Methods and Materials. All data generated or analyzed during this study are included in the manuscript and its additional files.

## Abbreviations

GIAD: Gastrointestinal adenocarcinoma
ESAD: Esophageal adenocarcinoma
STAD: Stomach adenocarcinoma
COAD: Colon adenocarcinoma
READ: Rectum adenocarcinoma
TGF-β: Transforming growth factor beta
CNV: Copy number variation
ssGSEA: Single sample gene-set enrichment analysis
DNN: Deep neural network
EMT: Epithelial mesenchymal transition
TMB: Tumor mutation burden
OS: Overall survival
PFI: Progression-free interval
DFS: Disease-free survival
SCNA: Somatic copy number alteration
