# Supplementary material for: Characterization of TGFβ-associated molecular features and drug responses in gastrointestinal adenocarcinoma
Source: BMC Gastroenterol. 2021 Jul 12;21:284. doi: 10.1186/s12876-021-01869-4 (PMC8274021; doi:10.1186/s12876-021-01869-4)
Supplement: Supplementary file 1 — Additional file 1. Supplementary figures. [file 12876_2021_1869_MOESM1_ESM.docx]

Characterization of TGFβ-associated molecular features and drug responses in gastrointestinal adenocarcinoma

Qiaofeng Zhang^1,2,3†^, Furong Liu^1,2,3†^, Lu Qin^4^, Zhibin Liao^1,2,3^, Jia Song^1,2,3^, Huifang Liang^1,2,3^, Xiaoping Chen^1,2,3^, Zhanguo Zhang^1,2,3*^, Bixiang Zhang^1,2,3*^

1. Hepatic Surgery Center, Tongji Hospital, Tongji Medical College, Huazhong University of Science and Technology, Wuhan, Hubei 430030, China.
2. Hubei Province for the Clinical Medicine Research Center of Hepatic Surgery, Wuhan, Hubei 430030, China.
3. Hubei key laboratory of Hepato-Pancreato-Biliary Diseases, Tongji Hospital, Tongji Medical College, Huazhong University of Science and Technology, Wuhan, Hubei 430030, China.
4. Department of Anesthesiology, Union Hospital, Tongji Medical College, Huazhong University of Science and Technology, Wuhan 430022, China.

† Qiaofeng Zhang and Furong Liu contributed equally to this work.

**Corresponding Authors**

Prof. Zhanguo Zhang, or Bixiang Zhang

Hepatic Surgery Center, Tongji Hospital, Tongji Medical College, Huazhong University of Science and Technology, Hubei Province for the Clinical Medicine Research Center of Hepatic Surgery, 1095 Jiefang Avenue, 430030 Wuhan, China. Tel.: +86 27 83663400; fax: +86 27 83662851; E-mail: zhanguo_tjh@hust.edu.cn, [bixiangzhang@163.com](mailto:bixiangzhang@163.com)

# Supplementary Figures


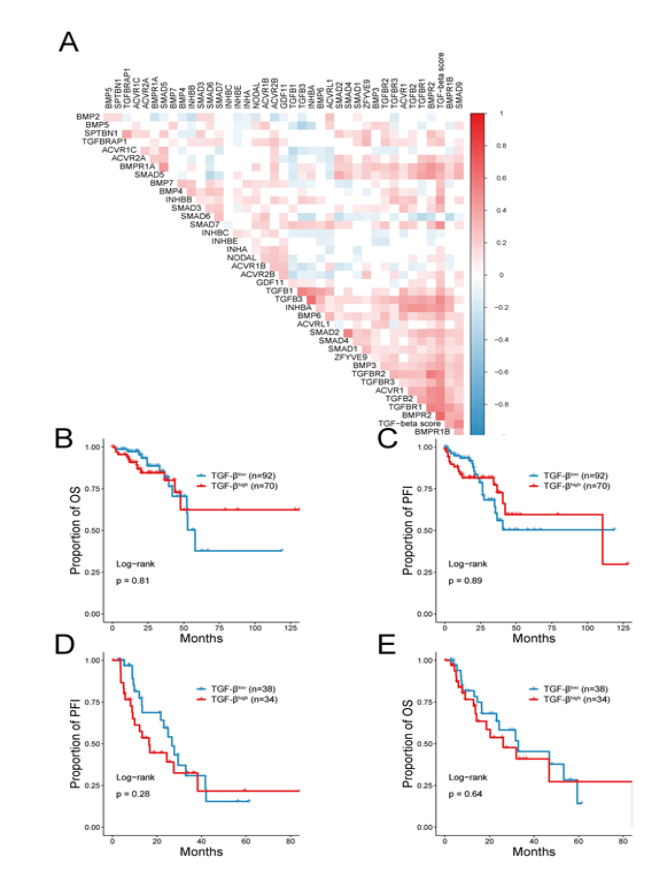


**Fig. S1.** Unsupervised classification of TGF-β status and prognosis in GIAD. **A** Correlation between TGF-β core genes and TGF-β score in GIAD. **B-E** Kaplan-survival curves (including overall survival and progression-free interval) for different TGF-β groups in READ (B for OS, C for PFI) and ESAD (D for PFI, E for OS). GIAD, gastrointestinal adenocarcinoma. READ, rectum adenocarcinoma. ESAD, esophageal adenocarcinoma.


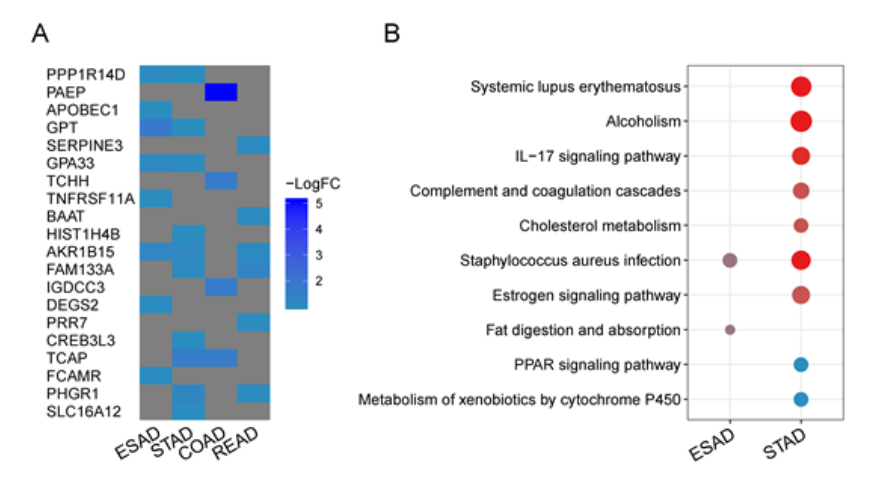


**Fig. S2.** TGF-β effects on mRNA and functional pathways. **A** The heatmap shows the most significantly down-regulated 20 mRNAs in the TGF-β­­^high^ group across each GIAD. **B** KEGG pathways enriched by significantly down-regulated mRNA in the TGF-β­­^high^ group (FDR < 0.05). GIAD, gastrointestinal adenocarcinoma. KEGG, Kyoto Encyclopedia of Genes and Genomes


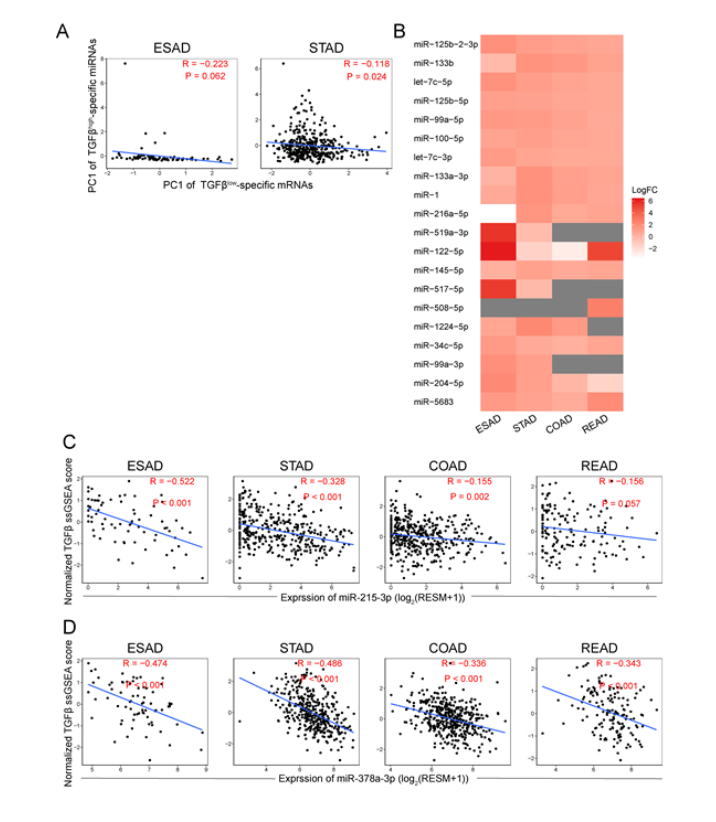


**Fig. S3.** Comparison of miRNA, and protein expression in different TGF-β status. **A** Correlation between the first principal component (PC1) of the expression of TGF-β­­^high^-specific miRNAs and their predicted targeted TGF-β­­^low^-specific mRNAs. (Among TGF-β­­^low^-specific mRNAs, mRNAs targeted by TGF-β­­^high^-specific miRNAs were only found in ESAD and STAD due to the restriction of mRNA number) **B** The heatmap shows the most significantly upregulated 20 miRNAs in the TGF-β­­^high^ group across each GIAD. **C-D** Negative correlation between miR-215-3p (and miR-378a-3p) expression and TGF-β score in 4 types of GIAD (Pearson correlation).


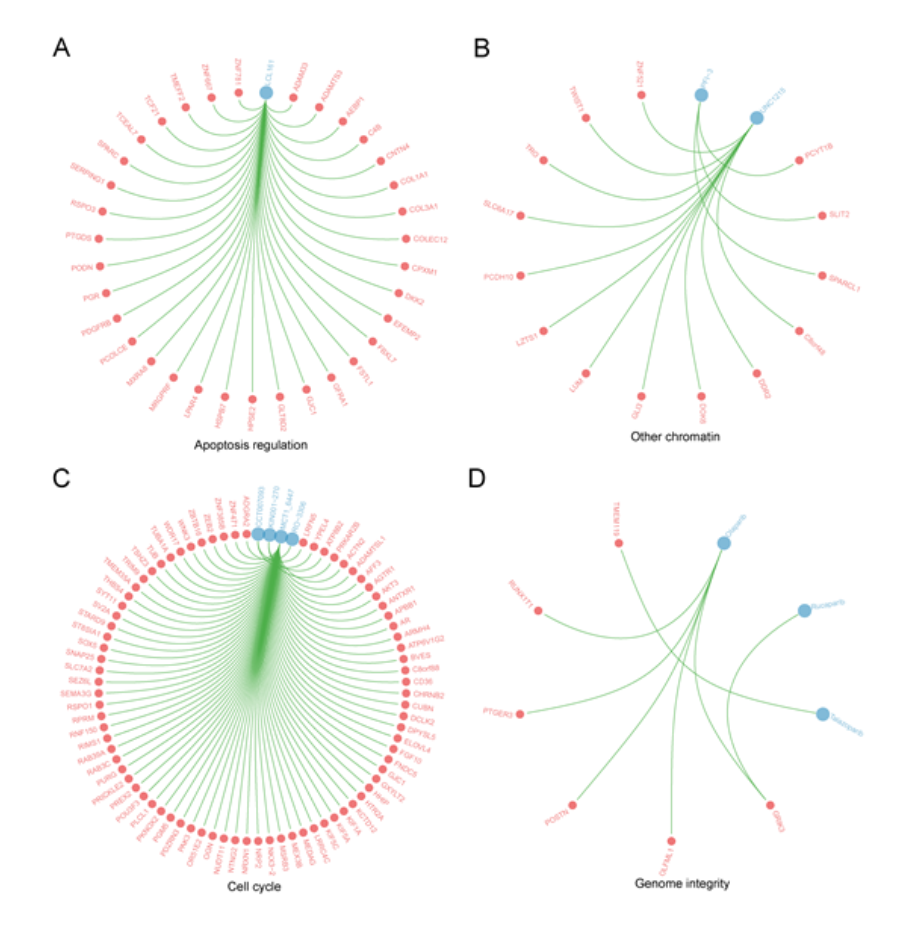


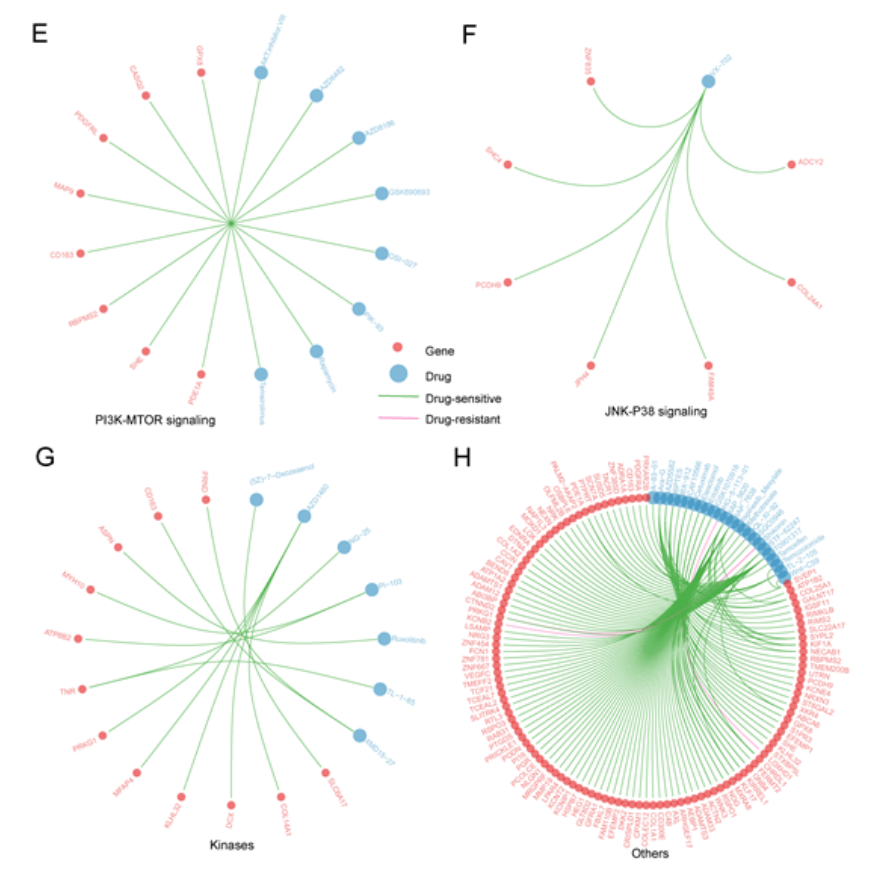


**Fig. S4.** TGF-β associated gene responses to drugs involved in different pathways; The red dots represent genes, the blue dots represent drugs, the green line indicates high gene expression is sensitive to drugs, and the pink line indicates high gene expression is resistant to drugs. **A** apoptosis, **B** other chromatin, **C** cell cycle, **D** genome integrity, **E** PI3K-mTOR signaling pathway, **F** JNK and p38 signaling pathway, **G** kinases, **H** other unclassified drugs.


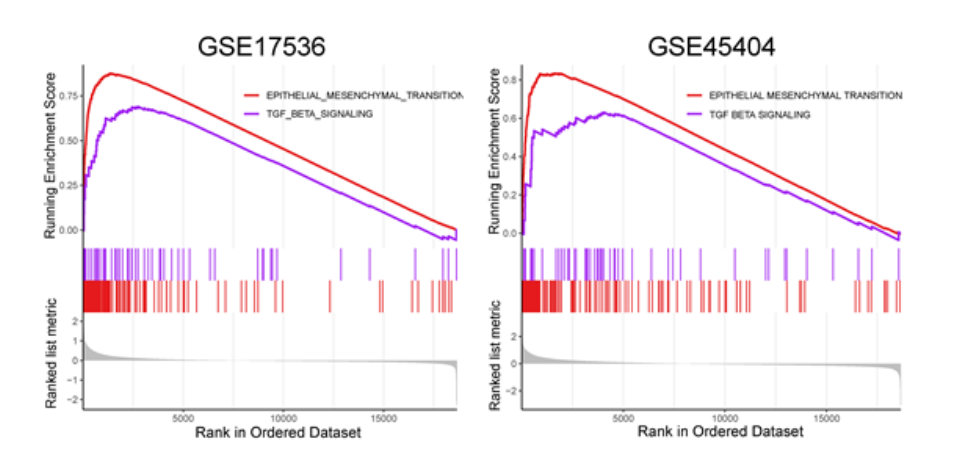


**Fig. S5.** Gene-set enrichment analysis showed that TGF-β and EMT pathways were significantly enriched in different TGF-β groups identified by deep neural network model in other datasets (GSE17536 (COAD), GSE45404 (READ)).


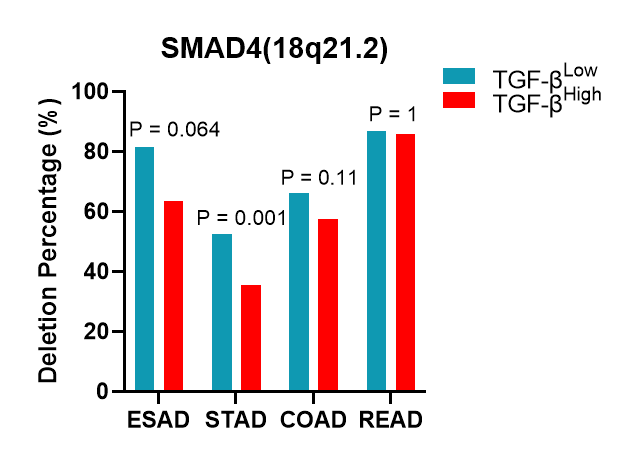


**Fig. S6.** Deletion of SMAD4 in different TGF-βgroups in gastrointestinal adenocarcinoma.
